# Effects of a Passive Lower-Limb Exoskeleton on Plantar Pressure Distribution and Postural Stability During Prolonged Occupational Standing: A Pilot Ergonomic Study

**DOI:** 10.3390/healthcare14131915

**Published:** 2026-07-01

**Authors:** Bianca Alina Achim Borza, Inés Martí Tripiana, Nadia Fernández-Erlingh, Juan Vicente Mampel, Benjamin Cuenca Valero, Javier Ferrer-Torregrosa

**Affiliations:** 1 Podiatry Department, Faculty of Medicine and Health Sciences, Valencia Catholic University San Vicente Mártir, 46001 Valencia, Spain; bianca.achim@mail.ucv.es (B.A.A.B.); ines.marti@mail.ucv.es (I.M.T.); nadia.fernandez@ucv.es (N.F.-E.); javier.ferrer@ucv.es (J.F.-T.); 2 Department of Physiotherapy, School of Medicine and Health Science, Catholic University of Valencia, 46001 Valencia, Spain; juan.vicente@ucv.es

**Keywords:** passive exoskeleton device, prolonged standing, plantar pressure, postural balance, occupational health

## Abstract

**Highlights:**

**What are the main findings?**

- The Chairless Chair^®^ 2.0 exoskeleton reduces peak and mean plantar pressures.
- Associated with lower COP displacement and velocity during prolonged standing tasks.

**What are the implications of the main findings?**

- Does not increase perceived fatigue and is well tolerated during simulated occupational tasks.
- Redistributes plantar loads and postural sway both in static and walking conditions.

**Abstract:**

**Background**: Prolonged occupational standing is associated with increased plantar pressure, impaired postural stability and fatigue, contributing to lower-limb musculoskeletal disorders. Passive lower-limb exoskeletons have been proposed as an ergonomic solution to reduce mechanical load without external power. **Objective**: To evaluate the effects of a passive lower-limb exoskeleton (Chairless Chair^®^ 2.0) on plantar pressure distribution, postural stability, perceived fatigue and user satisfaction in workers exposed to prolonged standing. **Methods**: A quasi-experimental pre–post study was conducted in 25 asymptomatic restaurant workers. Participants performed static standing and walking trials on a baropodometric platform with and without the exoskeleton. Plantar pressures were recorded in static and dynamic conditions and postural stability was assessed by centre-of-pressure (COP) displacement and velocity. Perceived fatigue was measured with the Borg CR10 scale, and usability and comfort with the QUEST 2.0 questionnaire. Repeated-measures ANOVA and non-parametric tests (*p* < 0.05) were applied. **Results**: Compared with standard footwear, the exoskeleton significantly reduced plantar contact area and peak and mean plantar pressures during standing and walking. Posturographic analysis revealed a significant effect of condition on COP-related variables, with significantly lower oscillation and velocity measures observed in the exoskeleton condition. Perceived fatigue remained low to moderate and did not differ significantly between tasks (*p* = 0.96). QUEST 2.0 scores reflected high perceived effectiveness, ease of use and comfort, although device weight was the least favourable characteristic. **Conclusions**: The Chairless Chair^®^ 2.0 improved plantar pressure redistribution and was associated with lower COP displacement and velocity values without increasing perceived exertion. The device was generally well accepted by participants, supporting its potential use as an ergonomic aid in occupations requiring prolonged standing.

## 1. Introduction

Prolonged standing is a prevalent requirement in numerous occupational environments, including manufacturing, healthcare, retail and the food service industry. Sustained upright postures have been associated with an increased risk of musculoskeletal disorders and circulatory problems, particularly affecting the lower limbs. Previous research has shown that prolonged plantar loading can lead to conditions such as plantar fasciitis, tendinopathies, venous insufficiency and pressure-related injuries [1], negatively affecting worker health, comfort and productivity [2,3]. These consequences may also translate into increased absenteeism and substantial economic costs for companies and healthcare systems.

One of the key biomechanical factors contributing to the development of lower-limb disorders during prolonged standing is the distribution of plantar pressures. When pressure is sustained or concentrated in specific foot regions over extended periods, functional alterations, discomfort and pain can occur, reducing workers’ operational capacity and increasing the likelihood of fatigue. Proper assessment of plantar pressure patterns is therefore essential for understanding biomechanical overload and identifying effective ergonomic interventions [1,4].

In this context, lower-limb exoskeletons have emerged as an innovative ergonomic strategy to mitigate the adverse effects of prolonged standing by reducing mechanical demands on the musculoskeletal system. Passive exoskeletons have gained attention due to their simplicity, affordability and independence from external power sources. These devices provide semi-sitting support, allowing workers to maintain neutral postures with lower muscular effort. Passive exoskeletons may also modify foot-loading dynamics by partially redirecting body weight through the mechanical structure of the device. By supporting part of the load at the pelvis and lower limbs, the exoskeleton may reduce the forces transmitted through the plantar surface and alter plantar pressure distribution. Evidence from previous studies suggests that passive exoskeletons can reduce muscle activation and perceived exertion in fixed postures [5,6], indicating potential value in occupational environments that require extended standing or constrained body positions.

Although recent literature has explored the mechanical and ergonomic benefits of passive devices, research specifically examining their influence on plantar pressure redistribution and postural stability is still limited. Some studies have highlighted that plantar pressure metrics can serve as valuable biomechanical indicators to evaluate assistive technologies, while others have proposed the use of plantar pressure information to optimise control strategies in exoskeletons [7,8]. Nonetheless, only a small number of investigations have focused on how passive lower-limb exoskeletons modulate foot loading patterns and centre-of-pressure (COP) behaviour during tasks representative of real occupational conditions. This gap is particularly relevant given that excessive plantar loading and impaired postural stability are well-established contributors to musculoskeletal strain and fatigue accumulation in workers exposed to prolonged standing [3].

From an occupational health perspective, assessing both plantar pressure distribution and postural stability provides valuable insight into the physical demands placed on the lower limbs during prolonged upright work. Variations in COP displacement, velocity and sway area are commonly used markers of postural control and fatigue, while changes in plantar pressure patterns can reveal compensatory strategies or mechanical stress concentrations. Understanding how passive exoskeletons influence these parameters is therefore critical to determining their true ergonomic value and feasibility for workplace implementation.

Based on the available evidence and the design characteristics of passive lower-limb exoskeletons, we hypothesized that the use of such a device during prolonged standing tasks would (1) reduce peak plantar pressures, (2) decrease COP displacement and velocity—indicating improved postural stability—and (3) maintain or reduce perceived exertion as measured with the Borg CR10 scale [9]. Accordingly, the aim of this study was to evaluate the effect of a passive lower-limb exoskeleton Chairless Chair^®^ 2.0 (Carl Stahl GmbH, Süßen, Germany) on plantar pressure distribution, postural stability, perceived fatigue and user comfort in workers exposed to prolonged standing, under both static and dynamic conditions.

## 2. Materials and Methods

### 2.1. Study Design

The present study followed a quasi-experimental, prospective pre–post design in which each participant was evaluated under two conditions: (1) performing prolonged standing and walking tasks with the passive lower-limb exoskeleton Chairless Chair^®^ 2.0 (Carl Stahl GmbH, Süßen, Germany) and (2) performing the same tasks without the device. Each participant served as their own control, following a within-subject repeated-measures approach to evaluate changes in plantar pressure distribution, postural stability, perceived fatigue and usability. The study protocol was approved by the Research Ethics Committee of the Catholic University of Valencia (code: UCV/2024–2025/030) and was conducted in accordance with the ethical principles of the Declaration of Helsinki [10,11]. It was registered at ClinicalTrials.gov under the identifier NCT06995469. Data collection was carried out between 1 March and 26 May 2025. All participants provided written informed consent prior to participation.

### 2.2. Participants

The sample consisted of 25 asymptomatic adult workers (18–60 years old), men and women in the restaurant industry. Restaurant workers were specifically selected because their occupational activities involve prolonged standing for a substantial proportion of the working day, making them an appropriate population for evaluating ergonomic interventions designed to reduce lower-limb loading. To participate, they were required to (i) have at least six months of experience performing work tasks involving prolonged standing, (ii) be physically able to perform their usual duties without limitations. On the other hand, individuals were excluded if they: (iii) were using orthopedic or ergonomic devices at the time of the study, such as insoles or special foot supports; (iv) presented uncontrolled medical conditions that could represent a risk to their health, such as uncontrolled hypertension; (v) were pregnant; or (vi) were unable to understand or adequately follow the instructions and procedures of the study.

### 2.3. Sample Calculation

The sample size was estimated to detect a moderate magnitude change in plantar pressures with and without exoskeleton, considering an expected effect size of 0.5, a significance level of 0.05 and a power of 80%. Under these assumptions, it was calculated that at least 21 subjects would be sufficient to achieve the desired power. Recruitment was done through direct contact with the collaborating company and initial interviews to ensure compliance with the inclusion criteria.

### 2.4. Intervention

The intervention evaluated the effects of the Chairless Chair^®^ 2.0 (Carl Stahl GmbH, Süßen, Germany) passive exoskeleton on workers undergoing controlled standing and walking tasks. First, a preparatory phase was conducted, in which participants were explained the purpose of the study, informed consent was obtained, and they were taught how to correctly position and adjust the device.

Before data collection, all participants completed a standardized familiarization session with the Chairless Chair^®^ 2.0 (Carl Stahl GmbH, Süßen, Germany). During this session, the device was individually adjusted according to each participant’s anthropometric characteristics and comfort requirements. Participants were instructed on how to walk, stand, and adopt the semi-seated posture provided by the device and were allowed to practice until they could perform the required tasks independently and safely.

The Chairless Chair^®^ 2.0 (Carl Stahl GmbH, Süßen, Germany) is a portable non-powered mechanical exoskeleton made of high-strength aluminum and technical polymers, weighing approximately 3.6 kg, capable of supporting up to 120 kg and adaptable to users from 1.50 to 2.00 m in height. It is attached to the body by means of a pelvic belt, thigh straps, shoe fastenings, and an adjustable harness. The seat height can be adjusted from 50 to 78 cm, providing a semi-seated posture that reduces the load on the lower extremities without limiting mobility.

The exoskeleton does not provide active assistance through motors or actuators. Instead, it operates through a passive mechanical load-transfer mechanism that redirects part of the user’s body weight from the pelvis and thighs to the ground through the structural frame of the device. During the semi-seated posture, the load is transmitted through the exoskeleton supports, partially bypassing the lower-limb musculoskeletal system and reducing the mechanical demand imposed on the plantar surface and lower extremities during prolonged standing tasks.

During the seated exoskeleton condition, participants adopted the semi-seated posture provided by the device while maintaining continuous bilateral foot contact with the pressure platform throughout data acquisition. This condition was included to characterize load redistribution under exoskeleton support and should not be considered biomechanically equivalent to unsupported standing, since the device partially transfers body weight through its structural supports and modifies the mechanical demands imposed on the lower limbs.

**Figure 1:**
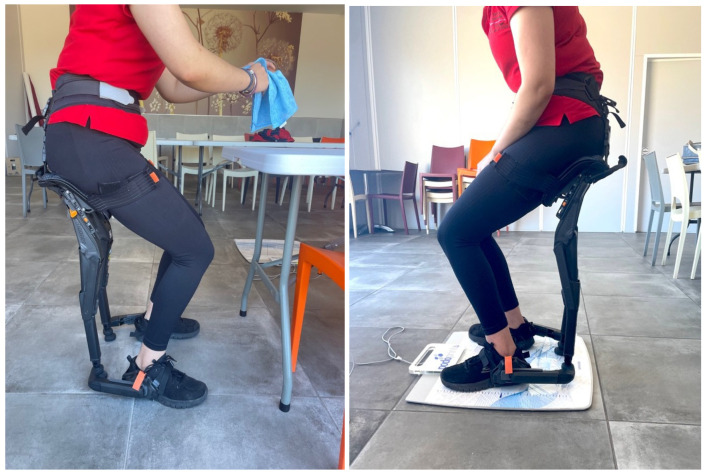
Experimental setup and testing conditions. (**Left**) Participant performing a simulated occupational task using the Chairless Chair^®^ 2.0 in the semi-seated configuration. (**Right**) Static assessment on the baropodometric platform for plantar pressure and centre-of-pressure (COP) measurements.

Each participant completed two testing conditions in a repeated-measures sequence. The complete experimental setup and testing conditions are illustrated in Figure 1. The baseline assessment was performed first without the exoskeleton, followed by the intervention assessment with the exoskeleton. In both conditions, the same assessment protocol was performed: (1) static assessment, consisting of quiet standing on a baropodometric platform to measure plantar pressure distribution and centre-of-pressure (COP) displacement and velocity; and (2) dynamic assessment, consisting of walking trials across the platform to record plantar pressure distribution during gait. Subsequently, participants performed their usual work tasks using the exoskeleton, and all measurements were conducted in a controlled environment following the same standardized procedure in both conditions to allow direct within-subject comparisons.

The experimental tasks were selected to simulate common occupational activities performed by restaurant workers during prolonged standing. Static tasks included glass-cleaning and food-preparation activities performed while maintaining an upright posture, whereas the dynamic task consisted of walking trials across the pressure platform. All participants completed the same tasks under standardized conditions and received identical instructions.

### 2.5. Study Variables

Plantar pressures (continuous quantitative): force exerted by body weight on the sole of the foot, measured in kilopascals (kPa) by means of a pressure platform, both dynamic and static.

Body sway (continuous quantitative): displacement of the COP in the anteroposterior and mediolateral plane, expressed in cm^2^.

Postural stability (continuous quantitative): ability to maintain balance, measured from the area and velocity of the COP.

Perceived fatigue (continuous quantitative): subjective level of exertion reported by participants, measured using the Borg CR10 scale (0–10) after each task to quantify perceived physical effort.

User satisfaction (ordinal quantitative): was assessed through the Quebec User Evaluation of Satisfaction with Assistive Technology [12] (QUEST 2.0). The questionnaire evaluates eight dimensions, including effectiveness, ease of use, comfort, weight, and overall satisfaction. Participants completed the questionnaire immediately after performing the tasks with the device.

### 2.6. Statistical Analysis

A within-subject repeated-measures analytical approach was used because each participant was assessed under the experimental conditions without and with the exoskeleton. Data normality was assessed using the Shapiro–Wilk test. Continuous variables with an approximately normal distribution were analyzed using repeated-measures analysis of variance (ANOVA). When the assumption of normality was not met, non-parametric paired comparisons were performed using the Wilcoxon signed-rank test. Post hoc comparisons were adjusted using the Bonferroni correction when appropriate.

Plantar pressure variables were analyzed separately for static and dynamic conditions. Static plantar loading variables included plantar contact area, force distribution, and weight distribution. Dynamic plantar pressure variables included plantar contact area, peak plantar pressure, and mean plantar pressure for each foot. Posturographic variables included medio-lateral and antero-posterior COP displacement, COP velocity, path length, and sway area.

Perceived fatigue scores obtained from the Borg CR10 scale were treated as ordinal repeated-measures data and compared across tasks using the Friedman test. Kendall’s W was calculated as an effect size for the Friedman test. User satisfaction assessed with the QUEST 2.0 questionnaire was analyzed descriptively using item-level means, standard deviations, medians, and overall satisfaction scores.

Effect sizes were reported where appropriate to support interpretation of the magnitude of the observed differences. Statistical significance was set at *p* < 0.05 for all analyses.

To explore the potential influence of anthropometric factors, exploratory Spearman correlation analyses were performed between body weight, body mass index (BMI), and the magnitude of plantar pressure reduction induced by the exoskeleton. Correlations were calculated for peak and mean plantar pressure variables, and statistical significance was established at *p* < 0.05.

## 3. Results

A total of 25 asymptomatic adult workers voluntarily participated in the study (age: 38.7 ± 14.6 years, height: 165.1 ± 8.5 cm, body weight: 70.6 ± 18.5 kg, body mass index: 25.8 ± 5.9 kg/m^2^) (see Table 1).

### 3.1. Influence of Body Weight and BMI

Exploratory correlation analyses were conducted to assess whether body weight and body mass index (BMI) influenced the magnitude of plantar pressure reduction observed with the exoskeleton. No statistically significant correlations were identified between body weight or BMI and changes in peak or mean plantar pressures (all *p* > 0.05). These findings suggest that the reductions in plantar pressure associated with the exoskeleton were generally consistent across participants with different anthropometric characteristics (Table 2).

### 3.2. Plantar Pressures and Load Distribution in Static

In the comparative analysis of the different experimental conditions (shoe, exoskeleton, and seated), differences were observed in the symmetry of plantar support variables between the left and right limbs. Surface area, force distribution, and weight distribution were analyzed separately because they represent distinct biomechanical outcomes measured on different scales.

**Figure 2:**
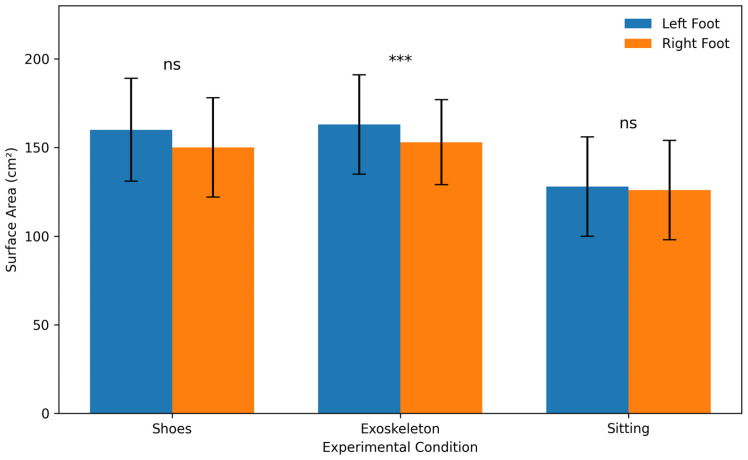
Left and right plantar contact area (cm^2^) under shoe, exoskeleton, and seated conditions during static assessment. Data are presented as the mean ± SD. Labels above the bars indicate the significance of left–right comparisons obtained from post hoc analyses: ns, not significant (*p* ≥ 0.05); *** *p* < 0.001.

In the shoe condition, no significant left–right differences were observed for surface area (mean difference = 10.32 cm^2^, *p* = 0.06), force distribution (mean difference = 3.92%, *p* = 0.06), or weight distribution (mean difference = 2.96 kg, *p* = 0.06), although small-to-moderate effect sizes were identified (see Figure 2).

**Figure 3:**
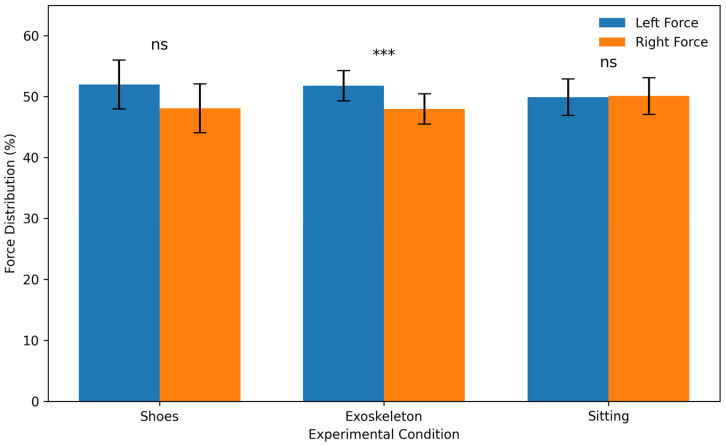
Left and right force distribution (%) under shoe, exoskeleton, and seated conditions during static assessment. Data are presented as the mean ± SD. Labels above the bars indicate the significance of left–right comparisons obtained from post hoc analyses: ns, not significant (*p* ≥ 0.05); *** *p* < 0.001.

In the exoskeleton condition, significant left–right differences were found for surface area (mean difference = 9.68 cm^2^, *p* < 0.001), force distribution (mean difference = 3.76%, *p* < 0.001), and weight distribution (mean difference = 2.72 kg, *p* < 0.001), suggesting a consistent asymmetrical load distribution while using the device (see Figure 3).

**Figure 4:**
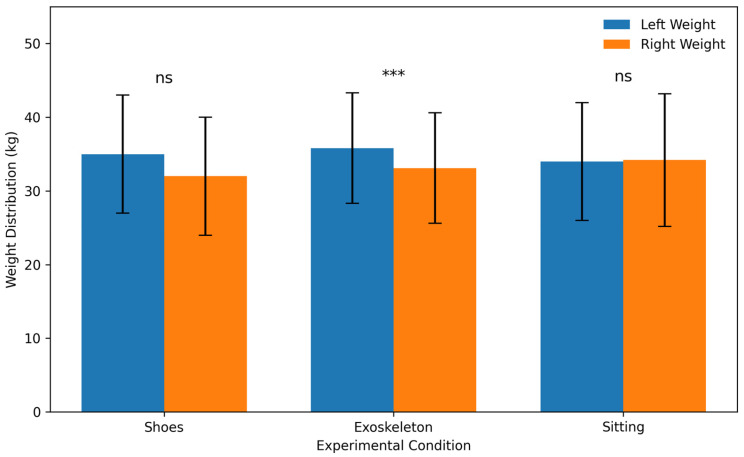
Left and right weight distribution (kg) under shoe, exoskeleton, and seated conditions during static assessment. Data are presented as the mean ± SD. Labels above the bars indicate the significance of left–right comparisons obtained from post hoc analyses: ns, not significant (*p* ≥ 0.05); *** *p* < 0.001.

In contrast, the seated condition showed minimal differences between limbs. Surface area (mean difference = 2.44 cm^2^, *p* = 1.00), force distribution (mean difference = −0.16%, *p* = 1.00), and weight distribution (mean difference = −0.16 kg, *p* = 1.00) demonstrated negligible effect sizes and no statistically significant asymmetry (see Figure 4).

Overall, the seated condition was associated with a more symmetrical distribution of plantar loading variables, whereas both the shoe and exoskeleton conditions exhibited greater left–right differences. These findings suggest that the semi-seated posture provided by the exoskeleton substantially modifies load distribution patterns compared with unsupported standing conditions.

The purpose of these analyses was to examine the symmetry of plantar loading between the left and right lower limbs within each experimental condition. These comparisons were performed to determine whether the exoskeleton modified bilateral load distribution patterns beyond the overall reductions in plantar pressure observed during standing. Therefore, Table 3 should be interpreted as a complementary analysis of load symmetry rather than as a direct comparison of exoskeleton versus non-exoskeleton conditions.

### 3.3. Posturographic Analysis

**Figure 5:**
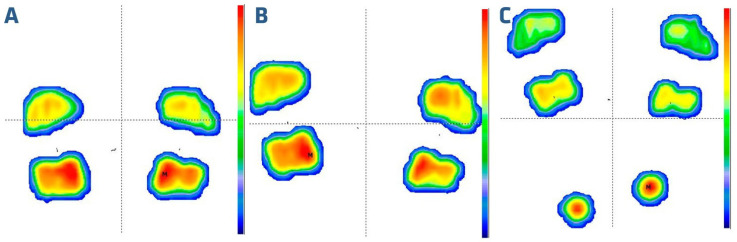
We observed the footprints with footwear in the conditions: (**A**) basal, (**B**) standing with exoskeleton, and (**C**) sitting with exoskeleton. The dashed vertical line represents the mediolateral axis (left = lateral, right = medial), and the dashed horizontal line represents the anteroposterior axis (top = forefoot, bottom = rearfoot). The color scale represents plantar pressure magnitude, ranging from low pressure (blue) to high pressure (red). The letter “M” indicates the medial aspect of the foot.

The descriptive analysis of the posturographic variables showed notable differences between the three experimental conditions: footwear, exoskeleton and seated (Table 4 and Figure 5). In terms of medio-lateral (L-M) oscillation, the exoskeleton presented the lowest values (1.59 ± 0.90 cm), followed by the footwear condition (2.20 ± 2.62 cm), while the seated exoskeleton condition showed larger COP excursions (5.64 ± 5.01 cm), reflecting altered support mechanics under the semi-seated configuration rather than unequivocal evidence of greater instability. This same pattern was observed in the medio- lateral velocity, where the exoskeleton showed lower velocity (0.81 ± 0.43 cm/s) compared to the footwear (0.96 ± 0.68 cm/s) and the seated position (2.50 ± 2.24 cm/s). Similarly, the anterior–posterior (A-P) oscillation and its velocity were smaller with the exoskeleton (3.08 ± 2.17 cm and 1.20 ± 0.71 cm/s) and with footwear (3.52 ± 2.28 cm and 1.26 ± 0.54 cm/s), while again sitting presented the highest values (5.12 ± 5.85 cm and 2.42 ± 2.54 cm/s). In addition, the parameters of path length and area traversed reflected this same effect: the exoskeleton reduced the length (7.99 ± 4.16 cm) and area (4.70 ± 5.36 cm^2^), compared to the footwear (8.51 ± 4.09 cm and 9.03 ± 20.11 cm^2^) and the sitting position, which showed a marked increase (18.97 ± 18.07 cm and 39.86 ± 68.79 cm^2^). The coefficients of variation indicated greater relative stability with the exoskeleton (0.42–0.70) and greater variability in the sitting condition (up to 1.73 in area traversed). These findings suggest that the exoskeleton may contribute to improved postural control during standing, as reflected by the lower COP oscillation and velocity values observed under the exoskeleton condition. However, the seated exoskeleton condition should be interpreted separately, as the larger COP excursions observed in this posture may reflect altered load transfer, partial unloading of body weight, and changes in foot-contact mechanics rather than a direct deterioration in postural stability.

### 3.4. Dynamic Analysis

**Figure 6:**
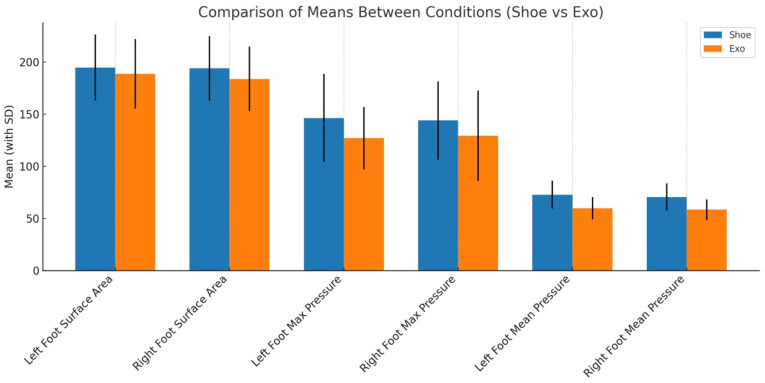
Comparison of means between conditions (shoe vs. exo).

Descriptive statistics for plantar pressure variables during gait are presented in Table 5 and Figure 6. During the dynamic evaluation of plantar pressures, significant differences were observed between the conditions with conventional and exoskeleton footwear, evidencing the impact of external support on load distribution during gait. The use of the exoskeleton consistently reduced the plantar support surface, both in the left (188.76 ± 33.38 cm^2^ vs. 194.68 ± 31.91 cm^2^) and right foot (183.80 ± 30.98 cm^2^ vs. 193.92 ± 30.93 cm^2^), suggesting better control of the base of support. Likewise, peak pressures decreased markedly with the exoskeleton, going from 146.28 ± 42.34 kPa to 126.92 ± 30.02 kPa in the left foot and from 143.88 ± 37.64 kPa to 129.20 ± 43.48 kPa in the right, while mean pressures were also significantly reduced (72.68 ± 13.39 kPa to 59.68 ± 10.75 kPa in the left foot; 70.48 ± 13.05 kPa to 58.24 ± 9.93 kPa in the right). Wilcoxon tests confirmed that these differences were statistically significant (*p* < 0.05), with moderate- to high-range biserial correlations (r = 0.51–0.93), indicating a relevant biomechanical effect. These findings suggest that the exoskeleton redistributes plantar loads during gait, resulting in lower peak and mean plantar pressure values compared with conventional footwear. This pattern is consistent with a reduction in plantar mechanical loading during walking under the experimental conditions evaluated.

### 3.5. Perceived Fatigue (Borg CR10 Scale)

**Figure 7:**
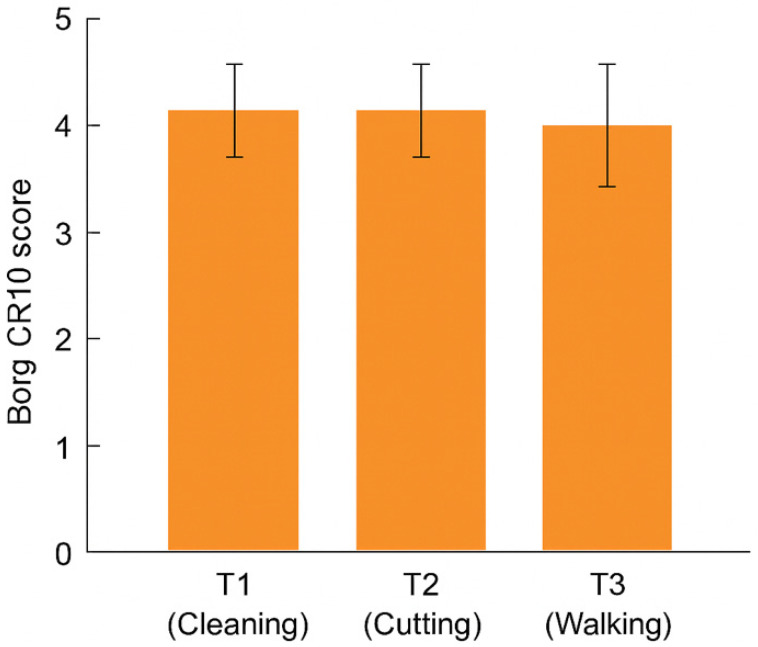
Perceived Fatigue (Borg CR10) Across Tasks.

The assessment of perceived fatigue using the Borg CR10 scale revealed that the two static tasks, cleaning a glass (T1) and cutting on a board (T2), were rated as moderately demanding, both with an identical mean score of 3.56 points. In contrast, the dynamic task of walking (T3) showed a slightly lower mean value (3.08 points), suggesting that participants perceived less exertion during movement. The standard deviation was high and comparable across all tasks (≈3 points), indicating substantial inter-individual variability. Some participants reported minimal perceived effort (0), whereas others reached the maximum imaginable exertion (10), even in tasks considered routine (Figure 7).

Quartile analyses further indicated that at least 50% of the sample rated the static tasks below 3 points, while the walking task demonstrated an even lower median (1.0). However, walking also showed a broader upper range (Q3 = 7), suggesting that for certain individuals, ambulation was perceived as demanding as the manual tasks. The Friedman test showed no statistically significant differences among the three tasks (χ^2^ = 0.087, *p* = 0.957), corroborated by a negligible Kendall’s W coefficient (W = 0.002), supporting the uniformity in fatigue perception.

These results highlight the importance of accounting for individual variability—likely related to physical conditioning, motor adaptation, or experience with the exoskeleton when interpreting perceived fatigue during different activities.

From an ergonomic perspective, although mean Borg CR10 scores were numerically lower during walking, no statistically significant differences were observed among the evaluated tasks (*p* = 0.957). Therefore, the present findings do not support a measurable effect of the exoskeleton on perceived fatigue under the experimental conditions studied. Nevertheless, the substantial variability in individual responses underscores the importance of personalized adjustments and adequate familiarization to ensure optimal device use. The low-to-moderate levels of perceived exertion reported across all conditions indicate that the exoskeleton was well tolerated by participants and did not increase subjective fatigue during task performance. Overall, these findings emphasize the importance of individualized evaluation when implementing occupational exoskeletons in workplace settings.

### 3.6. QUEST 2.0 Satisfaction Questionnaire

The QUEST 2.0 results showed a globally positive user perception of the exoskeleton, with particularly high scores in functional effectiveness and ease of use (see Table 6). In the safety and effectiveness subscale, item P4 (perceived safety) achieved a mean of 3.96, with both median and mode equal to 5, although with mild response dispersion (SD = 1.31). Item P8 (device effectiveness) received the maximum possible rating across all participants (mean = median = mode = 5; SD = 0), demonstrating unanimous satisfaction with the exoskeleton’s utility.

Regarding usability and fit, item P5 (general ease of use) also obtained a unanimous score of 5, while item P3 (fit/adjustment) showed a slightly lower mean of 4.24, indicating minimal variability in perceived adaptability. Comfort during use (item P7) was also highly rated (mean = 4.48; SD = 0.71). In the physical characteristics subscale, both dimensions (P1) and durability (P6) were well accepted (means of 4.24 and 4.48, respectively). The only item demonstrating notable variability was weight (P2), which yielded the lowest mean score (3.60) and the greatest dispersion, pointing to an area for potential technological refinement.

The overall QUEST 2.0 satisfaction score was 4.38/5, indicating a high level of user satisfaction with the Chairless Chair^®^ 2.0 passive exoskeleton. The highest-rated dimensions were ease of use and effectiveness (5.00/5), whereas weight received the lowest score (3.60/5). From a practical perspective, these findings suggest good user acceptance and support the feasibility of implementing the device in occupational settings requiring prolonged standing.

Collectively, the QUEST 2.0 findings indicate that the exoskeleton was perceived as an effective, ergonomic, and user-friendly device, with only minor aspects, such as weight and perceived safety for a few users, identified as potential targets for future improvements.

## 4. Discussion

The impact of passive lower limb exoskeletons on plantar pressures and postural biomechanics is a key element in both industrial and clinical applications. The results obtained in the present study with the use of the Chairless Chair passive exoskeleton^®^ 2.0 reveal significant positive effects in foot loading patterns and postural control, while perceived fatigue remained low and satisfaction with the device was generally high. These findings contribute to the growing body of evidence supporting the ergonomic relevance of passive exoskeletons in occupational contexts where prolonged upright postures are common.

Regarding plantar pressure, the exoskeleton condition resulted in reductions in peak and mean plantar pressures during both standing and walking, together with decreases in plantar contact area.

Although a reduction in plantar contact area would usually be expected to increase localized plantar loading, the simultaneous decrease in both contact area and peak and mean pressures suggests that part of the body load was transferred through the exoskeleton structure rather than being supported exclusively by the plantar surface. This load-bypassing mechanism may explain why plantar pressures decreased despite the smaller contact area.

The observed reductions in plantar pressure are biomechanically consistent with the load-transfer mechanism of the Chairless Chair^®^ 2.0. By providing structural support between the pelvis, thighs, and the ground, the device partially bypasses the lower-limb musculoskeletal system and reduces the amount of body weight transmitted through the plantar surface. Consequently, lower peak and mean plantar pressures can be achieved despite reductions in contact area.

These findings suggest that the semi-sitting support provided by the device facilitates a redistribution of load away from high-pressure plantar regions. Consequently, the observed reductions in plantar pressure are likely attributable not only to pressure redistribution within the foot but also to partial unloading of body weight through the exoskeleton structure. This pattern suggests a better redistribution of load and a possible reduction in mechanical stress on sensitive structures of the foot, which may help mitigate the risk of overuse injuries associated with prolonged standing.

Previous research has indicated that sustained or excessive plantar loading is related to discomfort, pain, and functional impairment in workers exposed to static postures [8], and that reducing localized pressures may represent a meaningful preventive strategy. The present results align with this perspective and complement prior studies showing reduced muscular demand when using passive exoskeletons in prolonged or constrained postures [13,14].

Given the well-established relationship between anthropometric characteristics and plantar loading, exploratory analyses were performed to investigate whether body weight and BMI influenced the magnitude of plantar pressure reduction observed with the exoskeleton. No statistically significant associations were identified between these variables and changes in peak or mean plantar pressures. These findings suggest that the biomechanical effects observed were relatively consistent across participants with different anthropometric characteristics. Nevertheless, the modest sample size of this pilot study may have limited the ability to detect weaker associations, and future studies with larger samples should further explore the potential influence of anthropometric factors on exoskeleton performance.

With respect to postural stability, COP displacement parameters (path length, sway area and velocity) were lower when participants used the exoskeleton compared with the standing condition without support. These findings are consistent with altered postural control under exoskeleton-assisted standing, characterized by lower COP displacement and velocity values compared with the unsupported standing condition [15,16]. Given that prolonged standing has been associated with impaired balance and increased postural oscillation in occupational settings [5], these results suggest that passive exoskeletons may help attenuate the gradual decline in postural control often observed during extended upright tasks. Moreover, prior investigations have highlighted the value of plantar pressure and COP-related metrics to examine the interaction between humans and assistive devices [7,17], further supporting the relevance of the present findings.

However, reduced COP displacement and velocity should not be interpreted exclusively as improved postural stability. In some contexts, reduced sway may also reflect a more constrained postural strategy, reduced ankle movement variability, or mechanical restriction imposed by the device. Therefore, the present COP findings should be interpreted as evidence of altered postural control under exoskeleton assistance rather than as unequivocal proof of improved balance.

It should also be noted that the seated exoskeleton condition represents a distinct biomechanical configuration and should not be interpreted as directly comparable to unsupported standing. The larger COP excursions observed under this condition may be influenced by the partial unloading of body weight and changes in support strategy produced by the exoskeleton. Consequently, these findings should be interpreted as reflecting altered postural mechanics rather than unequivocal evidence of reduced stability.

Regarding subjective perception, the Borg CR10 scores indicated that participants experienced moderate levels of exertion during the static tasks, with slightly lower values during walking. Although these scores suggest that the exoskeleton did not increase perceived effort, the absence of statistically significant differences between tasks reflects a relatively uniform perception of workload across activities [6,18]. This finding aligns with the notion that passive exoskeletons provide mechanical support without generating additional physiological burden, especially during prolonged standing [2]. The homogeneous distribution of fatigue ratings also suggests that the device did not introduce discomfort or excessive strain during task execution, supporting its acceptability in occupational contexts.

Finally, the QUEST 2.0 results reflected high levels of satisfaction, particularly regarding effectiveness, comfort and ease of use. Participants consistently rated the weight of the device as the least favorable attribute, a finding that parallels report in the literature noting that mass and bulkiness can influence perceived usability of assistive technologies [3]. Nevertheless, overall acceptance was positive, indicating that the exoskeleton is generally well tolerated and perceived as beneficial during prolonged standing tasks.

Overall, the combination of reduced plantar pressures, improved postural stability, stable fatigue levels and favorable usability ratings supports the potential ergonomic value of the Chairless Chair^®^ 2.0 in occupational settings. These results reinforce the role of passive exoskeletons as a feasible intervention for mitigating the biomechanical demands associated with prolonged standing [13]. Future studies with larger samples and longer exposure periods are warranted to verify long-term effects and to determine whether these benefits translate into reduced musculoskeletal symptoms or improved productivity.

Potential adverse effects associated with passive exoskeleton use should also be considered when interpreting the present findings. Although no serious adverse events were reported and overall user satisfaction was high, passive exoskeletons may introduce certain limitations that could influence biomechanical outcomes. Previous studies have described potential issues such as localized discomfort at attachment interfaces, restrictions in movement during specific tasks, altered gait patterns, asymmetrical load transfer, and increased perceived burden associated with device weight. In the present study, the weight of the device received the lowest score in the QUEST 2.0 questionnaire, suggesting that this characteristic may influence user experience despite the overall positive acceptance of the exoskeleton. Furthermore, the asymmetrical load distribution observed under some experimental conditions may reflect individual adaptation strategies to the device. Future studies should evaluate the long-term effects of passive exoskeleton use under real occupational conditions, including possible adverse biomechanical adaptations and their impact on comfort, performance, and compliance.

### Limitations of the Study

This study has several limitations that should be considered when interpreting the results. First, although the sample size was statistically sufficient to detect moderate changes in plantar pressure variables, it was relatively small and restricted to workers from a specific occupational setting, which may limit the generalizability of the findings to other populations and work environments. Second, the intervention was evaluated over a short period, without long-term follow-up, preventing assessment of potential adaptations to prolonged exoskeleton use, as well as possible cumulative effects on comfort, fatigue, or musculoskeletal function.

Additionally, although efforts were made to standardize all testing procedures, individual factors such as habitual footwear characteristics, physical conditioning, and previous exposure to prolonged standing tasks may have influenced biomechanical responses. Participants performed the assessments using their habitual occupational footwear in order to preserve ecological validity and reproduce real working conditions; however, differences in footwear characteristics may have contributed to inter-individual variability in plantar pressure outcomes. Furthermore, walking speed was not objectively controlled during the dynamic assessments, and participants were instructed to walk at their self-selected pace. Consequently, speed-related influences on plantar pressure distribution and gait biomechanics cannot be completely excluded. The study followed a fixed-order repeated-measures design in which all participants were assessed first without the exoskeleton and subsequently with the device. Consequently, potential order effects, learning effects, or task adaptation cannot be completely ruled out and may have influenced some outcomes. Future studies should incorporate randomized or counterbalanced testing sequences to strengthen internal validity.

As a pilot study, the biomechanical assessment focused primarily on plantar pressure and posturographic outcomes. Therefore, potentially relevant variables such as spatiotemporal gait parameters, ground reaction forces, joint moments, and objective physiological measures of fatigue were not evaluated. Future studies should incorporate these complementary assessments to provide a more comprehensive characterization of exoskeleton-related biomechanical adaptations.

Finally, the seated exoskeleton condition represents a distinct biomechanical configuration involving partial unloading of body weight through the exoskeleton structure. Therefore, this condition should not be considered directly comparable to unsupported standing. The posturographic and plantar-pressure responses observed under this condition likely reflect altered load-transfer mechanisms and support strategies rather than conventional standing balance behaviour, and should therefore be interpreted with caution.

## 5. Conclusions

The use of the Chairless Chair^®^ 2.0 passive exoskeleton proved effective in redistributing plantar loads and was associated with lower values in several posturographic parameters during standing tasks. Perceived fatigue remained low to moderate and did not differ significantly between experimental conditions. Participants reported good comfort and no significant discomfort, which reinforces its viability as an ergonomic solution. However, studies with larger sample sizes and prolonged follow-up are needed to confirm these findings and assess its applicability in different work environments.

## Figures and Tables

**Table 1:** Demographic characteristics of the sample.

	Total *n* = 25	Women *n* = 18	Men *n* = 7	*p*-Value
Age	38.68 ± 14.55	40.59 ± 14.52	34.63 ± 14.70	0.35
Foot size	39.48 ± 2.37	38.65 ± 2.00	41.25 ± 2.19	0.00
Weight (kg)	70.56 ± 18.52	66.76 ± 12.02	78.63 ± 27.14	0.14
Height (cm)	165.12 ± 8.53	162.76 ± 7.49	170.13 ± 8.90	0.04
BMI	25.77 ± 5.88	25.29 ± 4.99	26.79 ± 7.75	0.57

**Table 2:** Correlations between anthropometric variables and plantar pressure reduction induced by the exoskeleton.

Outcome Variable	Body Weight	*p*-Value	BMI	*p*-Value
Peak pressure reduction (left foot)	0.349	0.087	0.280	0.175
Peak pressure reduction (right foot)	0.187	0.371	0.184	0.379
Mean pressure reduction (left foot)	0.297	0.149	0.256	0.216
Mean pressure reduction (right foot)	0.263	0.204	0.329	0.108

Abbreviations: BMI, body mass index. Spearman correlation coefficients (ρ) are presented. No statistically significant associations were observed (all *p* > 0.05).

**Table 3:** Post hoc left–right comparisons of plantar contact area, force distribution, and weight distribution during static assessment under each experimental condition.

Condition	Variable	Left–Right Mean Difference	SE	df	t	Cohen’s d	pHolm
SHOE	Surface area (cm^2^)	10.32	4.37	24	2.36	0.61	0.06
SHOE	Force distribution (%)	3.92	1.63	24	2.40	0.23	0.06
SHOE	Weight distribution (kg)	2.96	1.19	24	2.49	0.17	0.06
EXOSKELETON	Surface area (cm^2^)	9.68	2.19	24	4.42	0.57	<0.001
EXOSKELETON	Force distribution (%)	3.76	1.00	24	3.75	0.22	<0.001
EXOSKELETON	Weight distribution (kg)	2.72	0.65	24	4.21	0.16	<0.001
SEATED	Surface area (cm^2^)	2.44	2.47	24	0.99	0.14	1.00
SEATED	Force distribution (%)	−0.16	1.17	24	−0.14	−0.009	1.00
SEATED	Weight distribution (kg)	−0.16	0.77	24	−0.21	−0.009	1.00

**Table 4:** Comparison of center-of-pressure (COP) variables across experimental conditions.

Variable	Footwear Mean ± SD	Exoskeleton Mean ± SD	Supported Sitting Mean ± SD
ML sway	2.20 ± 2.62	1.59 ± 0.90	5.64 ± 5.01
ML velocity	0.96 ± 0.68	0.81 ± 0.43	2.50 ± 2.24
AP sway	3.52 ± 2.28	3.08 ± 2.17	5.12 ± 5.85
AP velocity	1.26 ± 0.54	1.20 ± 0.71	2.42 ± 2.54
Path length	8.51 ± 4.09	7.99 ± 4.16	18.97 ± 18.07
Sway area	9.03 ± 20.11	4.70 ± 5.36	39.86 ± 68.79

Repeated-measures ANOVA demonstrated a significant effect of condition on postural stability outcomes (F = 6.020, *p* = 0.005, ω^2^ = 0.122). A significant Outcome × Condition interaction was also observed (F = 5.000, *p* < 0.001, ω^2^ = 0.116).

**Table 5:** Descriptive Statistics of Plantar Parameters in Dynamic Conditions (Footwear vs. Exoskeleton).

	n	Mean	SD	SE	Coefficient of Variation
Left foot surface (cm^2^) (SHOES DYNAMIC)	25	194.68	31.91	6.38	0.16
Left foot surface (cm^2^) (EXO DYNAMIC)	25	188.76	33.38	6.68	0.18
Right foot surface (cm^2^) (SHOES DYNAMIC)	25	193.92	30.93	6.19	0.16
Right foot surface (cm^2^) (EXO DYNAMIC)	25	183.80	30.98	6.20	0.17
Maximum P. Left Foot (kPa) (SHOES DYNAMIC)	25	146.28	42.34	8.47	0.29
Maximum P. Left Foot (kPa) (EXO DYNAMIC)	25	126.92	30.02	6.00	0.24
Maximum P. Right Foot (kPa) (SHOES DYNAMIC)	25	143.88	37.64	7.53	0.26
Maximum P. Right Foot (kPa) (EXO DYNAMIC)	25	129.20	43.48	8.70	0.34
Average P. Left Foot (kPa) (SHOES DYNAMIC)	25	72.68	13.39	2.68	0.18
Average P. Left Foot (kPa) (EXO DYNAMIC)	25	59.68	10.75	2.15	0.18
Average P. Right Foot (kPa) (SHOES DYNAMIC)	25	70.48	13.05	2.61	0.19
Average P. Right Foot (kPa) (EXO DYNAMIC)	25	58.24	9.93	1.99	0.17

**Table 6:** QUEST 2.0 item-level scores.

Item	Mean	SD	Median
P1 Dimensions	4.24	1.05	5
P2 Weight	3.60	1.26	4
P3 Adjustment/Fit	4.24	0.83	4
P4 Perceived safety	3.96	1.31	5
P5 Ease of use	5.00	0.00	5
P6 Durability	4.48	0.77	5
P7 Comfort	4.48	0.71	5
P8 Effectiveness	5.00	0.00	5

## Data Availability

The data presented in this study will be available on request from the corresponding author.

## Abbreviations

The following abbreviations are used in this manuscript:

ANOVA	Analysis of Variance
AP	Anteroposterior
BMI	Body Mass Index
COP	Centre of Pressure
CR10	Category Ratio 10 Scale
Df	Degrees of Freedom
kPa	Kilopascal
ML	Mediolateral
QUEST 2.0	Quebec User Evaluation of Satisfaction with Assistive Technology
SD	Standard Deviation
SE	Standard Error
ω^2^	Omega Squared Effect Size
Ρ	Spearman Rank Correlation Coefficient
χ^2^	Chi-Square Statistic
